# Wearable Real-Time Heart Attack Detection and Warning System to Reduce Road Accidents

**DOI:** 10.3390/s19122780

**Published:** 2019-06-20

**Authors:** Muhammad E. H. Chowdhury, Khawla Alzoubi, Amith Khandakar, Ridab Khallifa, Rayaan Abouhasera, Sirine Koubaa, Rashid Ahmed, Anwarul Hasan

**Affiliations:** 1 Electrical Engineering Department, College of Engineering, Qatar University, Doha-2713, Qatar; kalzoubi@qu.edu.qa (K.A.); amitk@qu.edu.qa (A.K.); rh1205366@student.qu.edu.qa (R.K.); ra1302696@student.qu.edu.qa (R.A.); sk1300288@student.qu.edu.qa (S.K.); 2 Department of Industrial and Mechanical Engineering, College of Engineering, Qatar University, Doha 2713, Qatar; rashid.ahmed@qu.edu.qa (R.A.); ahasan@qu.edu.qa (A.H.)

**Keywords:** heart attack, real time system, portable device, machine learning algorithm, support vector machine

## Abstract

Heart attack is one of the leading causes of human death worldwide. Every year, about 610,000 people die of heart attack in the United States alone—that is one in every four deaths—but there are well understood early symptoms of heart attack that could be used to greatly help in saving many lives and minimizing damages by detecting and reporting at an early stage. On the other hand, every year, about 2.35 million people get injured or disabled from road accidents. Unexpectedly, many of these fatal accidents happen due to the heart attack of drivers that leads to the loss of control of the vehicle. The current work proposes the development of a wearable system for real-time detection and warning of heart attacks in drivers, which could be enormously helpful in reducing road accidents. The system consists of two subsystems that communicate wirelessly using Bluetooth technology, namely, a wearable sensor subsystem and an intelligent heart attack detection and warning subsystem. The sensor subsystem records the electrical activity of the heart from the chest area to produce electrocardiogram (ECG) trace and send that to the other portable decision-making subsystem where the symptoms of heart attack are detected. We evaluated the performance of dry electrodes and different electrode configurations and measured overall power consumption of the system. Linear classification and several machine algorithms were trained and tested for real-time application. It was observed that the linear classification algorithm was not able to detect heart attack in noisy data, whereas the support vector machine (SVM) algorithm with polynomial kernel with extended time–frequency features using extended modified B-distribution (EMBD) showed highest accuracy and was able to detect 97.4% and 96.3% of ST-elevation myocardial infarction (STEMI) and non-ST-elevation MI (NSTEMI), respectively. The proposed system can therefore help in reducing the loss of lives from the growing number of road accidents all over the world.

## 1. Introduction

Fatal road accidents have become an alarming issue all over the globe. A driver with a medical condition is much more vulnerable to being hit and much more likely to cause a crash. This could injure the driver or anyone involved and can possibly be fatal [1]. Any disorder or condition that inhibits the driver’s strength, coordination, agility, mental capacities, judgment, attention, knowledge, or skill is a disorder or condition that can cause auto accidents. Some of the medical conditions that lead to accidents are seizures, strokes, heart attacks, impaired vision, or other conditions that include Alzheimer’s disease, Parkinson’s disease, and dementia [2]. Anything that inhibits any ability to drive creates a risk not only to the driver but to those sharing a road with them as well. 

Although acute medical illness is responsible for a small percentage of motor vehicle crashes, with estimates ranging from less than 0.1% to 3% in several studies [3,4,5], they are responsible for significant morbidity and mortality. In a study of 298 road crashes in the Adelaide metropolitan area [6], it was found that a medical condition was the main causal factor in 13% of the casualty crashes investigated and accounted for 23% of all hospital admission and fatal crash outcomes. A study among Japanese taxi drivers showed that a total of 98 drivers (23%) out of 844 experienced a collision or near miss incident due to their own acute health problems [7]. 

Heart attack is a sudden and sometimes fatal occurrence of coronary thrombosis, typically resulting in the death of part of a heart muscle. Heart attack is among the highest causes of human death and disability worldwide [8]. Even though heart attack is life threatening, it has early symptoms that could greatly help in saving many lives and avoiding consequences if it is detected and reported in a timely manner to the health care facilities. Therefore, to reduce road accidents that might result from the driver being precipitated by heart attack, there is an urgent need for a portable wearable system that can continuously monitor for any early symptoms of this medical situation, which could inform the patient (driver) as well as medical caregivers with the vehicle location. Thus, a driver could pull over the vehicle safely before losing his/her consciousness to avoid potentially fatal consequences, and medical caregivers could arrive and provide lifesaving procedures to rescue the driver in a timely manner. 

**Figure 1:**
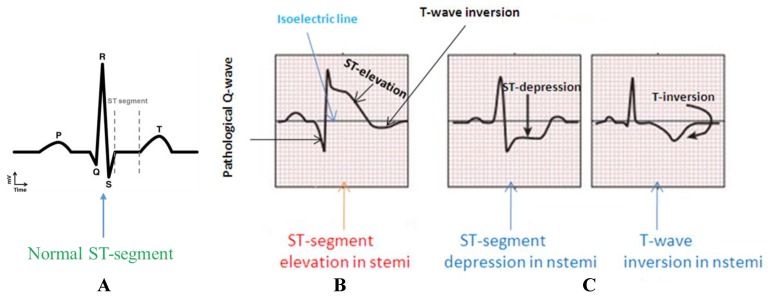
Comparison of the ST-segment variations in a normal subject (**A**) and in MI patients with ST-elevation myocardial infarction (STEMI) (**B**) and non-ST-elevation MI (NSTEMI) (**C**).

Myocardial infarction (MI), commonly known as heart attack, is a serious medical emergency in which the supply of blood to the heart is suddenly blocked, usually by a blood clot in the coronary artery [9,10]. A lack of blood to the heart may seriously damage the heart muscle and can be life threatening. There are three types of heart attack—ST-elevation myocardial infarction (STEMI), non-ST-elevation myocardial infarction (NSTEMI), and coronary spasm [11]. Electrical signals recorded from the heart are referred to as electrocardiograms (ECG or EKG). A normal ECG trace (Figure 1A) consists of components that indicate electrical events during one heartbeat. P wave is the first short upward movement of the ECG tracing, which indicates that the atria are contracting and pumping blood into the ventricles. The QRS complex normally begins with a downward deflection, Q, a larger upwards deflection, a peak (R), and then a downwards S wave. The QRS complex represents atrial repolarization and ventricular depolarization and contraction. The PR interval indicates the transit time for the electrical signal to travel from the sinus node to the ventricles. T wave is normally a modest upwards waveform representing ventricular repolarization.

A STEMI (Figure 1B) occurs when a coronary artery becomes completely blocked and a large portion of the muscle stops receiving blood, whereas NSTEMI (Figure 1C) is due to partial blockage of the coronary artery [12]. MI symptoms vary among individuals; around 89.7% have chest pain, and 67.4% have pain in the upper part of the left arm [13]. One major symptom other than extreme sweating is the irregularity of the ECG pattern. A STEMI will cause the ST complex to be elevated; however, this is not the case in NSTEMI (https://myheart.net/articles/nstemi/). STEMI is detectable and more lethal; therefore, it is the main concern [12].

The existing ambulatory ECG monitoring systems take a considerable amount of time and effort to record ECG signals in patients through long-term hospitalization, and the ECG data have to be sent to professionals for diagnostic analysis. However, a wearable ECG device can help in real-time monitoring of heart attack because it can make decisions itself by observing irregular events of ECG signals and identification of sudden heart attack and will be particularly useful for drivers in saving their lives and avoiding accidents.

To observe the changes in ECG patterns, the ECG signal needs to be acquired, amplified, filtered, and analyzed for MI detection through various algorithms. There are recent studies that suggest either the development of a two-electrodes based amplifier alone [14,15] or a three-electrode wearable portable ECG system [16,17]. In [18], two gel-less electrodes-based ECG systems were designed for low power portable application to acquire ECG signals and heart rate while the subject was engaged in different physical activities. In the two-electrode design, there was a reference electrode compensation circuitry to avoid saturation and to increases common mode rejection. It also had baseline correction and isolated ground, which helped in direct current (DC) drift and common mode noise illumination. This system was useful for the two-electrode based signal acquisition. However, this system did not include any machine learning algorithm to detect any abnormality of the heart. In [19], two Ag/AgCl electrodes were used for ECG signal acquisition. The ECG signal was then transmitted at 2.4 GHz band to a personal computer (PC). The system was capable of operating for 49 h continuously from a rechargeable lithium-ion battery. This system was also designed to monitor ECG signal without a smart algorithm. Yap et al. [20] presented a chest-belt type two-electrode wireless real-time ECG recording system, which was based on an android monitoring application. This design included motion artifacts compensation and R-peak detection for ECG arrhythmia detection. The experiment results showed significant improvement of R-peak detection accuracy during fast movement activity states. However, the MI detection was not implemented in this research. The algorithm implemented in [21] detected any ST elevation and compared it to an isoelectric line. Each R peak was extracted by comparing to a threshold of 0.6. The T peak was located between the R-peak with a margin of 400 ms and the J point (beginning of the ST-isoelectric line) with a margin of 80 ms. This approach was implemented in this research as a reference work for linear classification to evaluate its robustness and suitability for the driver application. Another linear classification and threshold-based algorithm was developed in [22]. The algorithm used LabVIEW Mobile Module and Bluetooth for receiving data. Both systems were capable of real time analysis of the ECG signal. However, they were not suitable for MI detection. Using the Physiobank MI database, Sopic et al. [23] showed a support vector machine (SVM) based real-time MI detection system with a classification accuracy of 90%. An enhanced and optimized adaptive filter with optimal filter coefficients selection and a fuzzy rule-based algorithm was shown to resolve the motion artifact issue in the two-electrode small size wearable chest belt ECG system mounted with a three-axis accelerometer for ECG and ubiquitous activity recording in daily life. However, the authors did not mention any abnormality classification algorithm in this work [24].

Cardiovascular disease is the leading cause of medical illness and sudden death in commercial motor vehicle drivers (CMV) [25]. Cardiovascular disease has an increasingly powerful impact on the health and safety of CMV drivers because of its prevalence in the population, its progressive nature, the aging work force, and recent advances in diagnosis and therapy. Most studies have shown that cardiovascular disease is the major cause of acute medical illness that results in motor vehicle crashes [4,26,27,28]. However, a study [29] from the National Motor Vehicle Crash Causation Survey (NMVCCS) in the USA reported that 95% of the drivers in crashes precipitated by medical emergencies experienced seizures (35%), blackouts (29%), diabetic reactions (20%), or heart attack (11%) prior to the crashes, where heart attack was shown to contribute to 11% of the crashes. A National Transportation Safety Board (NTSB) study [30] described fatal heavy trucks crashes where 19 out of 185 truck drivers were fatally injured. Seventeen of those 19 crashes (89%) involved a form of cardiac incident at the time of the accident, e.g., sudden incapacitation of the driver due to an acute heart problem [31].

In this work, the authors proposed the development of a prototype model for a wearable real-time heart attack detection and warning system to be used by a vehicle driver. The authors worked on an accurate detection of symptoms of a heart attack event using a machine-learning algorithm. The system is continuously monitoring the ECG trace of the driver, and if any pre-symptom of heart attack is found, the driver is informed to pull over his/her vehicle, and an alerting call and message with the patient location is sent to a pre-defined number to inform emergency medical ambulance facilities. In this manner, a driver can save himself/herself by avoiding fatal road accidents before losing his/her consciousness, and emergency medical services can approach the driver in a timely manner to provide required lifesaving medical procedures to avoid any life-threatening consequences. Accordingly, the proposed system will help in controlling the growing number of road accidents all over the world. Furthermore, as the proposed system is wearable and portable, any person with previous history (or even without previous history) of heart attack can take advantage of this system in different settings (e.g., work, home, driving, etc.). Last but not least, this system can help the vehicle driver in claiming insurance facility if the accident is caused due to the driver’s ill health. 

The rest of the paper is divided into five sections. Section 2 discusses the research methodology and the details of the studies done in this work. Section 3 shows a detailed analysis of the studies followed by the results discussed in Section 4 and finally concluded in Section 5.

## 2. Experiment Details and Methods

**Figure 2:**
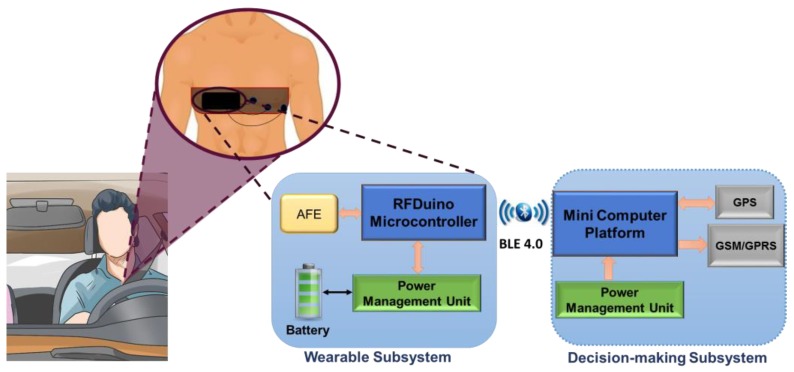
Overall system block diagram.

The prototype system consists of two subsystems that communicate wirelessly using Bluetooth low energy (BLE) technology—a wearable sensor subsystem and an intelligent heart attack detection and warning subsystem, as shown in Figure 2. The sensor subsystem uses dry ECG electrodes to sense the electrical activity from the chest area to produce an ECG trace. The raw signals from the patient’s body are sent continuously through the Bluetooth interface to the detection and warning subsystem. The later continuously processes and analyzes the raw measurements to detect any symptoms related to heart attack. 

Real-time ECG signal acquisition, amplification, filtering, digitization, and wireless transmission are accomplished by the wearable sub-system. This subsystem is attached to a chest belt to be worn by a driver. This includes three dry electrodes (reference and two electrodes for differential acquisition), an analogue front end (AFE), and an RFduino microcontroller with an embedded BLE module. Dry electrodes acquire the potential difference from the body, amplify and filter it through AFE, and then digitize and transmit the raw data to the decision-making subsystem. Reusable and smaller dimension dry electrodes (Cognionics, Inc) are embedded in a chest belt to be worn by a vehicle driver. The AFE is required to maintain high signal-to-noise ratio (SNR), high common mode rejection, and fewer baseline drift and saturation problems. AD8232 (analog devices) AFE is an integrated signal conditioning module to extract, amplify (60 dB gain), and filter (bandwidth 0.48–41 Hz) the ECG signal in the presence of noisy conditions. The module includes lead-off detection, single supply operation, adjustable gain control, rail-to-rail output, a three-pole adjustable low pass filter (LPF), a two-pole adjustable high pass filter (HPF), and an integrated right-leg drive (RLD). A 50 Hz center frequency Wien bridge notch filter is used to remove the 50 Hz line frequency from the ECG signal. The output of the notch filter is digitized using RFduino and transmitted to the decision-making subsystem.

**Figure 3:**
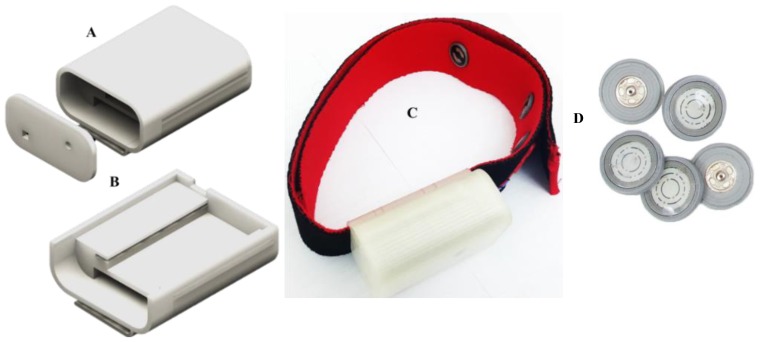
Exterior (**A**) and interior view (**B**) of the three-dimensional (3D) model of the electrocardiograms (ECG) amplifier and (**C**) the ECG amplifier with the chest best and (**D**) dry electrodes.

The ARM Cortex M0 is the core of RFduino microcontroller and has a built-in Bluetooth 4.0 low energy module. RFduino uses Arduino integrated development environment (IDE) as user interface program, which allows testing and running of pre-written sketches and takes advantage of the existing libraries. RFduino has a 10-bit analog-to-digital (ADC) module, which is capable of acquiring the ECG signal at a 500 Hz sampling rate with the resolution of 2.93 mV. Moreover, the dimension, the low-power consuming feature, the 3.0 V operating voltage, and the built-in BLE module make RFduino an excellent choice for this application. The wearable subsystem is powered through a lithium (Li)-ion battery that is connected directly through a power cell board. The power management module (PMM) is a boost converter (to 3.3 V and 5 V) and micro-universal serial bus (USB) charger in one. The boost converter is based on the TPS61200 from Texas Instrumentation (TI) and has a solder jumper selectable at 5 V and 3.3 V outputs and an under-voltage protection of 2.6 V. The module can be charged by a mobile charger using an on-board micro-USB connector and is capable of delivering 3.3 V or 5 V. The PMM is configured to provide 3.3 V output to the RFduino, the AFE module, and the notch filter. The wearable module is a chest-belt with an ECG amplifier, the packaging of the ECG amplifier is designed using a three-dimensional (3D) printer, and the assembly is shown in Figure 3.

**Figure 4:**
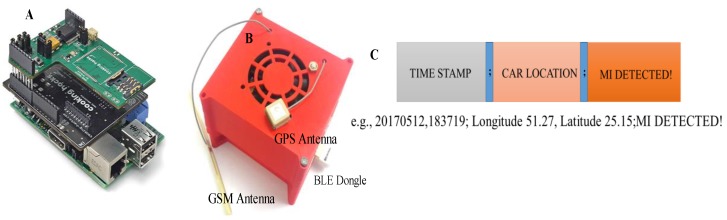
Intelligent decision-making subsystem hardware (**A**) with the 3D printed model (**B**) and the packet format used for communication (**C**).

The intelligent heart attack detection and warning subsystem is the brain of the whole system and plays a major role in system operation. This module detects the event of heart attack in real-time depending on the acquired ECG signal and the trained machine learning model using the Massachusetts Institute of Technology-Beth Israel Hospital (MIT-BIH) ST Change Database [32]. This subsystem is built around the single board computer, Raspberry Pi 3 (RPi3) (quad core 1.2 GHz, 1 GB RAM, microSD, WiFi, BLE). The ECG signal is received in RPi3 over the BLE interface from RFduino. ECG data are buffered for 10 s, and the baseline drift is corrected, segmented to ECG-beats (one ECG trace), and smoothened using a digital filter. This subsystem incorporates SIM 908 global system for mobile communications (GSM)/General Packet Radio Service (GPRS) and the Global Positioning System (GPS) module interfaced directly to RPi3 using the cooking-hack shield. The shield is popular for its low power consumption feature during GSM communication and GPS data acquisition. The shield is directly fitted on the Raspberry Pi 3 and powered by the RPi3 power. The standard DC connector to supply electrical power for portable accessories used in cars is used to power the decision-making module. The shield uses a serial communication interface for its data communication with RPi3. However, the hardware serial port of the RPi3 is dedicated to its internal BLE module. Therefore, the internal BLE of the RPi3 has to be disabled to establish a communication between the RPi3 and the SIM908 module. The RPi3 runs in autonomous startup login mode and a python script is started in startup, which is used to acquire and buffer the ECG data in the local RPi3 memory. Figure 3 and Figure 4 show the various blocks of the prototype wearable and decision-making system. Details of the detection algorithm are discussed in the analysis section. 

### 2.1. Study 1: Hardware Performance Evaluation

All the experimental studies were carried out on healthy male and female volunteers with the ethical approval from the local ethical committee. In order to evaluate the performance of the designed hardware, the following experiments were conducted for the wearable and decision-making systems:

#### 2.1.1. Evaluation of the Wearable System

Three experiments were conducted using the wearable system: i) dry electrodes performance, ii) lead configurations, and iii) ECG system performance.

The quality of the ECG signal acquired by the prototype model in the vehicle environment using both dry and conventional Ag/AgCl wet electrodes while the user was driving the vehicle at different speeds was evaluated to compare the performance of the electrodes.

The driver was constantly controlling steering, thus hands movement made it impossible to extract ECG from the left arm (LA), the right arm (RA), and the right leg (RL). Electrodes had to be placed in such a place that the driver’s natural driving would not be hampered while preserving the normal ECG signal. To select the right electrode placement area that was convenient for the driver and at the same time had high noise immunity, experiments were conducted using dry electrodes for testing the performance of the different lead configurations in the vehicle environment at different speeds.

**Figure 5:**
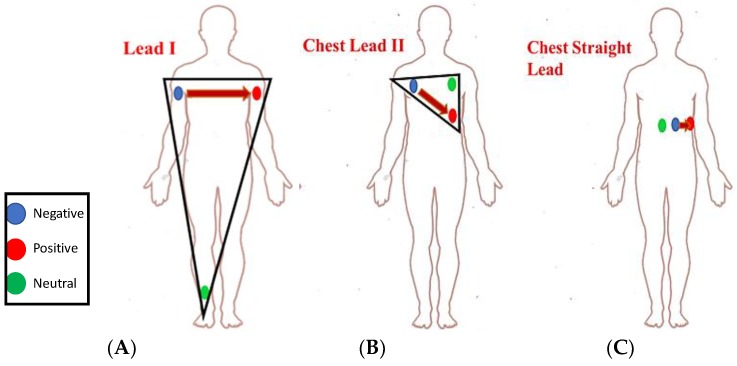
Different ECG lead configurations tested: (**A**) Lead I; (**B**) Chest Lead II; (**C**) Chest Straight Lead.

There were three lead configurations tested using three ECG electrodes, as shown in Figure 5:

(i) Lead I ECG Recording: ECG sensors were placed on the right arm (RA), the left arm (LA), and the right leg (RL), as shown in Figure 5A.
(ii) Chest Lead II ECG Recording: Figure 5B shows a commonly used three-electrodes chest ECG recording configuration where the electrodes were placed on the upper left torso in a triangular shape.
(iii) Chest Straight Lead: Figure 5C is a commonly used configuration in magnetic resonance imaging (MRI) environments that are less susceptible to motion and vibration artifacts, which any user might experience inside the vehicle.

ECG signals were acquired from two healthy subjects by placing electrodes simultaneously in the chest area for the prototype and a commercial wireless wearable ECG amplifier (BioRadio) [33] to compare the quality of the ECG traces.

#### 2.1.2. Decision-Making and Alerting System

The SIM 908 modules functionality of short message service (SMS), call origination and reception, and GPS data acquisition were tested initially in the command-line interface of the RPi3 using attention (AT) commands. Multi-threaded Python code was written for RPi3 using the arduPi library [34] developed by a cooking hack to automatically initiate an SMS text if the abnormality in ECG trace was detected. Experiments were carried out to evaluate the performance of the alerting system, which included an audible (buzzer) local alert for the driver and the GSM/GPRS based call and SMS alerts to the pre-defined number.

#### 2.1.3. Reliability of the BLE Transmission between Two Sub-Systems

Experiments were conducted to check the performance of the wireless transmission system in transmitting the ECG data over the wireless interface and to evaluate the fidelity of the signal at a 500 Hz sampling frequency. RFduino uses BLE protocol to transmit data through a Generic Attribute Profile (GATT) to RPi3. GATT is the standard BLE devices communication services and characteristics protocol. Before any BLE connection is established, the device should advertise itself. Advertising and connection processes are controlled by the Generic Access Profile (GAP). Once a connection is initiated, the peripheral device (wearable subsystem’s RFduino) can only be connected to one central device (RPi3). In our system, RFduino was the GATT master, which held the GATT service and characteristic. On the other hand, the GATT client (RPi3) was responsible for sending requests and receiving responses. To ensure reliable data transmission to the RPi3 without missing any data packet, notification of incoming data packet reception in the BLE buffer was used. Moreover, to increase the sampling frequency of the RFduino to 500 Hz in data acquisition, ECG data were buffered before transmission, and after every 20 ms, a buffered frame was sent to RPi3. This was to ensure low power consumption of the wearable system while keeping high frequency sampling for reliable ECG acquisition. The RFduino timer interrupt was used to ensure 2 ms interrupt-driven data acquisition to guarantee the 500 Hz sampling frequency.

#### 2.1.4. Power Consumption of the Two Subsystems

**Figure 6:**
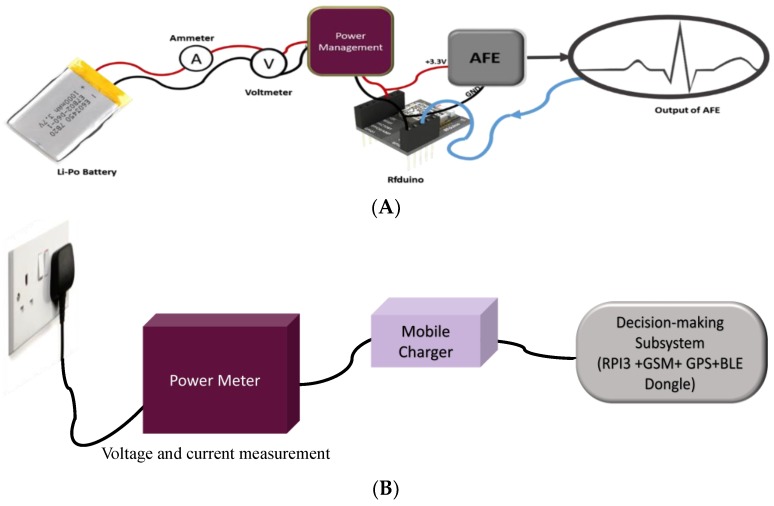
Set-up of power consumption study for wearable subsystem (**A**) and decision-making subsystem (**B**).

A detailed study was accomplished on the power consumption of the two subsystems to compare the efficiency of the system in terms of the power consumption. Overall power consumption of the wearable system was tested in four test scenarios: low-power consumption mode of RFduino, ECG signal acquisition with no BLE transmission, ECG acquisition and burst BLE transmission, and continuous ECG acquisition and BLE transmission with a 500 Hz sampling of ECG data while the frames of buffered ECG data were transmitted every 20 ms. Figure 6A shows the complete power consumption testing arrangement for the power management section of the wearable subsystem.

The decision-making subsystem was designed with multiple components—RPi3, GPS, a GPRS/GSM shield, a cooling fan, and a BLE dongle. To characterize the current consumption of the complete subsystem, individual system component consumption was evaluated by the test set-up shown in Figure 6B. The power meter was drawing 3.2 mA without connecting it to the RPi3, which represented the power consumption of the mobile charger and the power meter itself. This mobile charger was connected to RPi3 using RPi’s micro-USB port. Different scenarios of current consumption were tested to find out the current consumption by the decision-making module in these cases. We tested how much current it consumed during the initialization process, the BLE data transmission and processing, idle mode, fan off mode, GSM initialization mode, and active transmission mode of GSM and GPS.

### 2.2. Study 2: Performance Evaluation of MI Detection Algorithms

In order to evaluate the performance of the MI detection algorithms, the authors experimented with a linear classification algorithm in the preliminary study and then 22 different machine learning (ML) algorithms [three decision tree, two discriminant analysis, six SVMs, six k-nearest neighbor (KNN), and five ensembles classifiers] were trained for real-time ECG classification. Experiments were done in two phases. In the first phase, we used a public labeled database “MIT-BIH ST Change Database” for identifying suitable classification algorithms for detecting heart attack. In this database, normal healthy subject data and abnormal MI patient data are available. The MIT-BIH ST change database has 28 ECG recording datasets in total, where 14 subjects’ ECG recordings were normal ECG traces, seven patients had T-inversion, and the other patients experienced long-term recordings that showed ST elevation and depression. The databases are developed and managed by the Physiobank organization. Physiobank is a database of “well-characterized digital recordings of physiologic signals and related data for the use by the biomedical research community”. Therefore, training and testing of the machine learning algorithm were accomplished by real healthy patient and MI patient datasets. In the second phase of real-time implementation in the RPi3, the wearable system was tested on normal subjects and an ECG simulator to simulate abnormal ST-elevated MI situations to test the functionality of the complete system in real-time.

The linear classification algorithm was implemented in the PC (Intel core i7, 8 GB RAM, Windows 7 x64) environment using Matlab 2015b. We used sliding windows of size 10 seconds to include several consecutive interbeat intervals. The sliding window continuously moved to the next interbeat interval, overlapping half of the interval. Therefore, the features for abnormality could be extracted from the sliding window. Twenty-two machine learning algorithms were implemented initially in the PC environment using Matlab 2015b to classify MI. The two best performing algorithms were identified, and then the best performing algorithm was implemented in RPi3 using Python 2.7. Signal pre-processing was accomplished using signal processing, wavelet transformation, and statistics and machine learning toolboxes in the Matlab on the PC using Numpy (v1.13.3), Matplotlib (v3.0.2), PyWavelets (v0.5.0), and LIBSVM Python libraries in Python on RPi3.

#### 2.2.1. Linear Classification of MI

**Figure 7:**
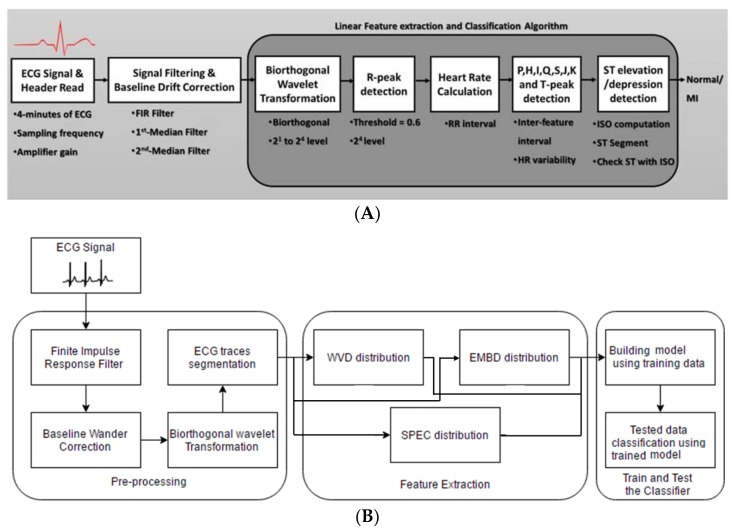
Blocks of the linear classification (**A**) and machine learning (ML) (**B**) based MI detection algorithm.

**Figure 8:**
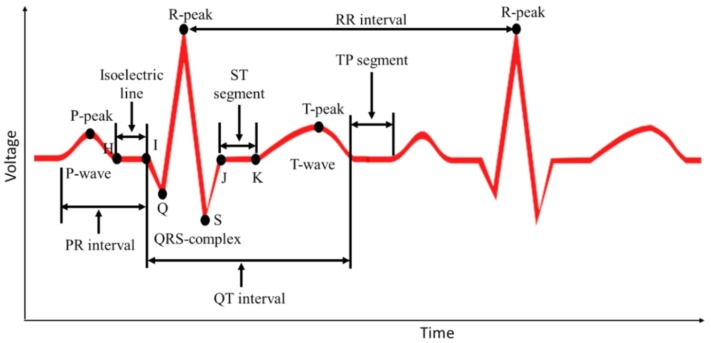
Normal ECG waveform with the waves and intervals. Note: user-defined HI line represents the isoelectric (ISO) line and JK line represents the ST segments.

The linear classification detected three major deflections in the ECG signals: P and T waves along with a QRS complex. The ECG signal underwent several signal processing steps—filtering, baseline wonder removal, and wavelet transformation—before the deflections were detected, which is explained in analysis (Section 3). The blocks for the linear classification MI detection algorithm are shown in Figure 7A. Finding the R wave and detecting its peak was the most important part in this method in order to diagnose heart rhythm abnormalities. All other ECG parameters were estimated based on this value. The R-peak, where the heartbeat had the maximum amplitude, was extracted by setting the threshold level approximated by 0.6. The basic idea of this algorithm was taken from the Tompkins method [35]. Taking into consideration that the QRS complex duration was almost 60 ms and knowing the exact time where the R peak occurred, the S and the Q points could be easily detected. These points should have been within the interval of approximately 32 ms after and before the R-peak, respectively, where the first minimum points took place. Following the same method and the same timing interval, the J point was estimated to be the first point reaching zero, taking the negative S point as a reference. The T peak was allocated by using information to indicate precisely the right interval in which the T peak should have occurred. Hence, the T peak was the maximum point between 400 ms from the R peak and 80 ms after the J point. It was detected by comparing neighboring points and searching for the maximum value in this restricted interval. Consequently, the K point was estimated to be in a duration of 35 ms from T peak. The ECG signal could be treated symmetrically, which eased the detection of the work by dealing with the ECG trace in a similar manner after and before the R peak, which was executed to get the Q, the K, and the P peak as well as the H point. This helped to identify the changes occurring in the ST segment, and any change that occurred in the ST segment was essential in identifying the heart attack, as it was continuously compared to the isoelectric line (as shown in Figure 8) to spot any abnormality in the case of ST elevation, depression, or T-inversion, which represented myocardial infarction.

#### 2.2.2. ML Algorithm-Based Classification of MI

The blocks for the ML algorithm based MI detection algorithm are shown in Figure 7B. Some of the pre-processing steps of linear classification were common for ML based classification. It was used in segmenting the ECG signal into ECG-beats. R peaks were used as the most visible features of the complete ECG trace, while T peaks and P peaks demonstrated the boundaries of each ECG trace. The length of each trace differed from patient to patient with the presence of the inter-subjective and intra-subjective variability between the patients. Five hundred traces from each patient from the MIT-BIH database with either normal or abnormal heart rhythms were generated. Several time (t)-domain, frequency (f)-domain, and time–frequency (t,f) domain features were extracted from the segmented ECG data, which are detailed in the analysis section. Three popular quadratic time–frequency distributions (QTFDs)—Wigner–Ville distribution (WVD), Spectrogram (SPEC), and extended modified B-distribution (EMBD)—were compared in this work. The (t,f) features could be obtained by extending t-domain and f-domain features to the joint (t,f) domain. The performance of each feature in detecting ST elevation or T-wave inversion in ECG was evaluated by performing an area under the curve (AUC) analysis on the values of the features extracted from the ECG segments belonging to different abnormal cases (e.g., ST elevation and T-inversion). Five-fold cross validation was used for training and validation of the 22 different machine learning models. The best performing model was used to classify the two-class MI detection problem.

## 3. Analysis

Discussed below are the several pre-processing steps that were common to both classification algorithms as well as some that were specific to the ML algorithm. 

### 3.1. Pre-Processing Steps

The pre-processing steps of the ECG signal used in both classification algorithms are discussed below:

*Digital Filtering:* It was essential to filter the signal and eliminate the inherent noises, which is commonly called baseline wander and can be initiated by respiration, body movements, or even perspiration, as well as by power line interference of 50 Hz. The ECG signal was prone to muscle noise (EMG), bowel movements (EGG), and noise generated from electroencephalography (EEG) [36]. In addition, ECG was often contaminated by artifacts constituted through electrodes or the interference of the signal processing hardware [37]. Thus, it was essential to use a filtering technique along with baseline wander correction for further analysis of the signal for better feature extraction of the ECG signal. Finite impulse response filter (FIR) was selected using the window method to smooth the noisy signal by slicing the array of data into selected length windows, computing averages of the data within that range, and maintaining the process throughout the data set using the moving-average filter [38].

**Figure 9:**
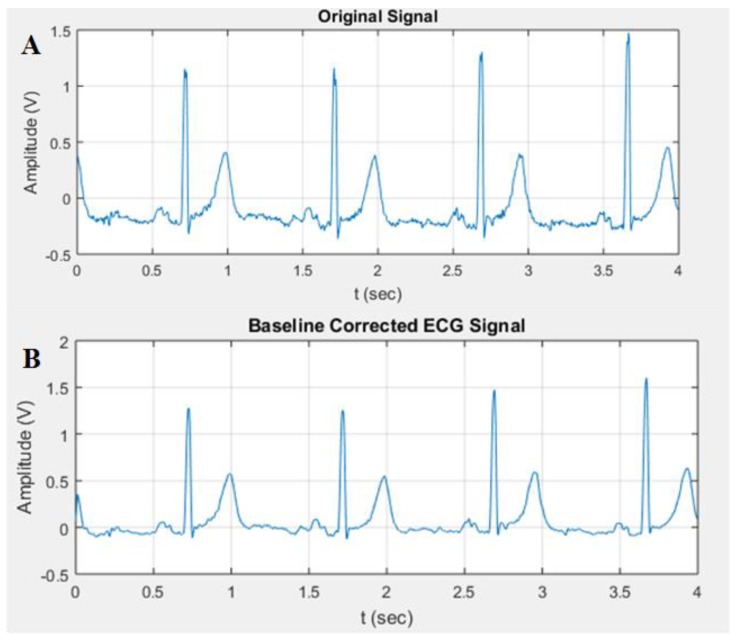
Overall representation of the signal before and after baseline wander correction: (**A**) Original signal; (**B**) baseline corrected ECG signal.

*Baseline Wander Correction:* It was noticed that the baseline of the signals was not exactly at zero level. This made the isoelectric line not well defined for extraction and computation, which resulted in inaccurate MI detection. Thus, baseline wander correction was required. The signal was fed into a 200 ms width median filter to eliminate QRS complexes and P-waves. The obtained signal from the filter was processed with a median filter of 600 ms to eliminate the T-waves. The attained signal was then subtracted from the resultant signal of the FIR filter. The plots shown in Figure 9 represent the signal before and after baseline correction. 

*Biorthogonal Wavelet Transformation:* Continuous wavelet transformation was used for synthesizing the ECG signal, and this allowed us to inspect how the frequency component varied within certain ranges of time, i.e., how the ECG wavelets were generated. The frequency of the QRS complex was mainly present in the 2^3^ and the 2^4^ scales. Since the scale 2^4^ showed less noise compared to 2^3^, in this work, scale 2^4^ was used for R-peak detection.

### 3.2. Linear Classification

All the ECG points and time intervals were calculated, and heart rate (HR) and isoelectric (ISO) potential and ST segment potential were calculated and compared with each other to spot any abnormality in the case of ST elevation, depression, or T-inversion, as mentioned in the preliminary study section. Classification accuracy was calculated for normal and noisy data.

### 3.3. ML Based Classification

In the subsequent section, we discuss the remaining signal pre-processing steps, features extraction, training and validation of the SVM model, classification, and the steps to implement the SVM to detect MI in a real-time manner.

**Figure 10:**
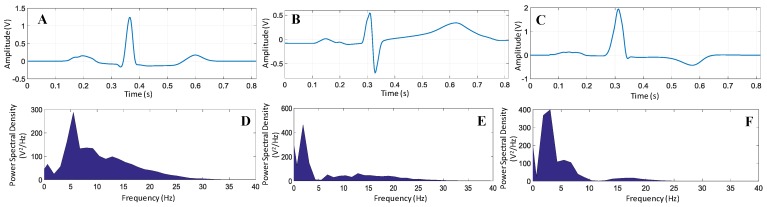
ECG trace averaged over traces and its power spectral density for (**A**,**D**) normal, (**B**,**E**) ST-elevation, and (**C**,**F**) T-wave inversion, respectively.

*Segmentation:* To compensate the variability of the ECG trace length, zeroes were padded at the beginning and at the end of any trace that had less than the trace-length defined in the segmentation process. Segmenting the ECG signals into traces allowed for data manipulation and permitted primary observation of the unique features of each type. Figure 10 shows the difference in the ECG signal in both time and frequency domains for normal and abnormal conditions in which the abnormality was considered to be divided in two types, T-inversion and ST elevation. In order to have accurate and general presentation of each category (normal, ST-elevated, and T-inverted), 3500 ECG traces over 28 different subjects were considered. Therefore, the total number of ECG traces considered in this study was 10,500. We averaged the ECG traces for each different case where each one was a combination of different subjects to overcome the inter-patient variability problem due to typical varying parameters across the patients [32].

*Features Extraction:* The power spectral of the signal in Figure 10D,E,F shows that the power spectral density peaks appeared at different frequencies for normal and abnormal ECG signals. Moreover, the power spectral density rapidly vanished and crossed zero for both affected cases. This was not the case for non-affected ECG, as frequencies appeared up to 30 Hz. This reflected that the simple frequency domain feature could help in classifying the ECG signals. However, t-domain, f-domain, and (t,f)-domain provided insight into the signal while compensating for the noise or motion artifacts.

Mean, variance, skewness, kurtosis, and coefficient of variation were used as t-domain features to spot abnormalities in the ECG-beats. The mean described the average value for the readings, the variance showed how much the recorded signal deviated from the mean, skewness showed the T wave symmetry in shape, and kurtosis showed the degree of peakness of the T wave shape. The coefficient of variation described the relationship between data points based on the dispersion around the mean value. This permitted the comparison of data points for the data series that had different mean values. Table 1 summarizes the t-domain features with discrete ECG beat and x[n] as an N-point signal.

Frequency-domain features such as the spectral flux, the spectral entropy, and the spectral flatness were employed for the detection of abnormalities in ECG signals. The spectral flux measured the rate of change of the spectral content of the ECG signal with time, whereas the spectral flatness (SF) measured the level of uniformity of the energy distribution in the frequency domain and was defined as the geometric mean of the magnitude spectrum of the ECG signal normalized by its arithmetic mean. However, spectral entropy (SE) measured the randomness in the distribution of the signal energy in the frequency domain.

Time- and frequency-domain features extended to produce joint (t,f)-features. Wigner–Ville distribution (WVD), Spectrogram (SPEC), and extended modified B-distribution (EMBD) were used to extend the (t,f)-features. Table 2 shows the t-domain, the f-domain, and the (t,f)-domain features extracted [39] from the ECG traces. These were used for training the ML models and testing the ECG data using the trained best performing model.

*Performance Evaluation:* The Receiver operating characteristic (ROC) analysis was used to evaluate the performance of the ML algorithm for classification. It explained the capability of the trained model in classifying different classes. The higher the value of area under the curve (AUC) was, the better the model was in distinguishing the normal beat from the abnormal. The performance of each feature in detecting ST elevation and T-wave inversion in ECG was evaluated by performing an ROC analysis on the values of the feature extracted from ECG segments belonging to different abnormal cases (e.g., ST elevation and T-wave inversion).

Apart from the AUC value, confusion matrix and several standard statistical evaluation parameters were used to evaluate the performance of the algorithms:

True Positive Rate (TPR)/Recall/Sensitivity:

(1) $$Recall=\;\frac{TP}{TP+FN}$$

Specificity:

(2) $$Specificity=\frac{TN}{TN+FP}$$

False Positive Rate (*FPR*):

(3) $$FPR=1-Specificity=\frac{FP}{TN+FP}$$

Precision:

(4) $$Precision=\;\frac{TP}{TP+FP}$$

F-measure or score:

(5) $$F\;score=\frac{2*Recall*Precision}{Recall+Precision}$$

Accuracy:

(6) $$Accuracy\;\left(ACC\right)=\frac{TP+TN}{Total\;positives\;and\;negatives}$$

where *TP* is true positive, *TN* is true negative, *FP* is false positive, and *FN* is false negative. 

The above-mentioned parameters were estimated using five-fold cross validation such that the entire database was divided into five equal sets. Out of five sets, four sets were used for training, while one set was used for testing. This process was repeated five times such that each set was tested once. The final results were obtained by averaging the results of all the iterations. The averages of recall, specificity, precision, f-score, and accuracy were calculated for the four iterations along with their standard deviation. Performance evaluations of three different time-frequency distributions (TFDs) were calculated to identify which TFD produced higher accuracy.

### 3.4. Real-Time Implementation 

Two models were trained and validated using the labeled (training and testing) data; one was for the STEMI (MI for ST segment elevation), and the other model was for the NSTEMI (MI for T-wave inversion). A Python-based machine learning algorithm was implemented using the open source LIBSVM library [40], and multi-threaded application was used for real-time pre-processing and classification of ECG data. The decision of the real-time classifier was updated every 10 seconds, and the alerting tone was generated locally to the driver. The in-house built C++ program was used to initiate an emergency call and text short message using the GSM/GPRS module to a pre-defined emergency number. 

## 4. Results and Discussion

This section summarizes the results from the hardware experiments and algorithm performance evaluation studies.

### 4.1. Hardware Performance Evaluation

**Figure 11:**
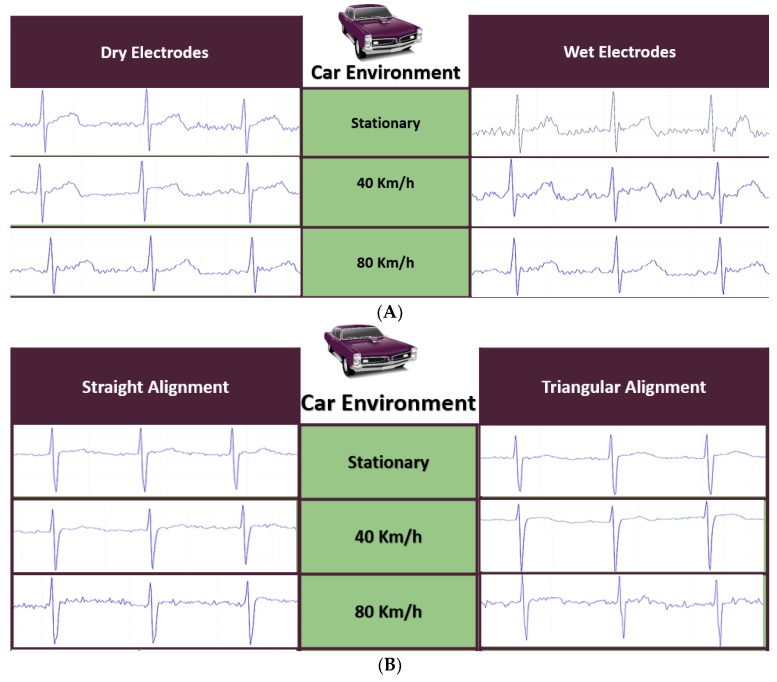
Performance of dry electrodes in comparison to wet electrodes (**A**) and different lead configurations (**B**) at different vehicle speeds.

The ECG signal acquired from dry electrodes was found comparable to the wet electrodes. Moreover, dry electrodes are reusable and non-disposable, which reduces the running cost of the system. In addition, the conductivity of the dry electrode increases over time, which guarantees signal quality in a real-time system. However, in wet electrodes, after a couple of hours, the gel starts to dry, impedance increases, and signal quality becomes poor. Figure 11A shows the ECG traces for different driving scenarios for both type of electrodes. It was observed that the ECG signals were continually getting better in the case of dry electrodes, which was not the case in wet electrodes. It is worth mentioning that the wearable system was robust enough to acquire the ECG signal from the driver during the different driving speed scenarios.

**Figure 12:**
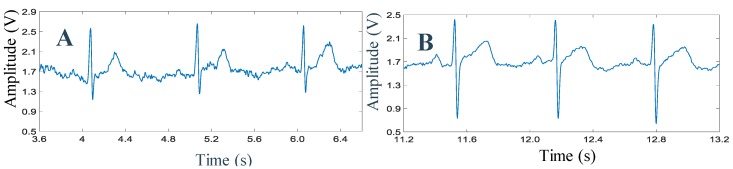
ECG data collected wirelessly from (**A**) subject 1 and (**B**) from subject 2.

Figure 12 shows the ECG traces recorded from two different subjects chosen randomly. The quality of the ECG signal acquired wirelessly shows the evidence of the performance of the prototype system and the reliability of the wireless communication unit used in the prototype.

Initial trials revealed that the Lead I configuration (RA, LA, and RL) was not practical for the driver and was significantly affected by noise due to hand movements during controlling the steering; the comparison between the configurations (ii) and (iii) is shown in Figure 11B. Generally, both configurations produced similar quality ECG signals and were comparable to clinical grade ECG traces, even at higher vehicle speeds. However, the selection of the configuration then needed to be based on how comfortable it would be for the driver. From the results, it was evident that the straight alignment configuration of the electrode was the best option for ECG acquisition in the driving environment.

The ECG signal sent from the wearable system to the RPi3 was logged and compared for the transmission reliability. It was compared frame by frame to identify any discrepancy of the received data (e.g., packet loss) during the transmission. It was observed that acknowledged and frame-based data transmission ensured the communication reliability, and no packet loss was observed in the transmission.

The SIM 908 GSM module was used for calling and sending SMS to the medical emergency service with the car location information acquired using the GPS module embedded in the SIM 908. During normal ECG acquisition, pre-processing, and classification, GPS data were not acquired to keep the power requirement of the RPi3 minimum. GPS data were only acquired when any abnormality was detected; then, time stamp, latitude, and longitude of the car location with the MI detection information were sent. The ECG simulator was connected to the wearable system to generate normal and abnormal ECG signals. It was evaluated whether the system could detect real-time ECG abnormality. 

**Figure 13:**
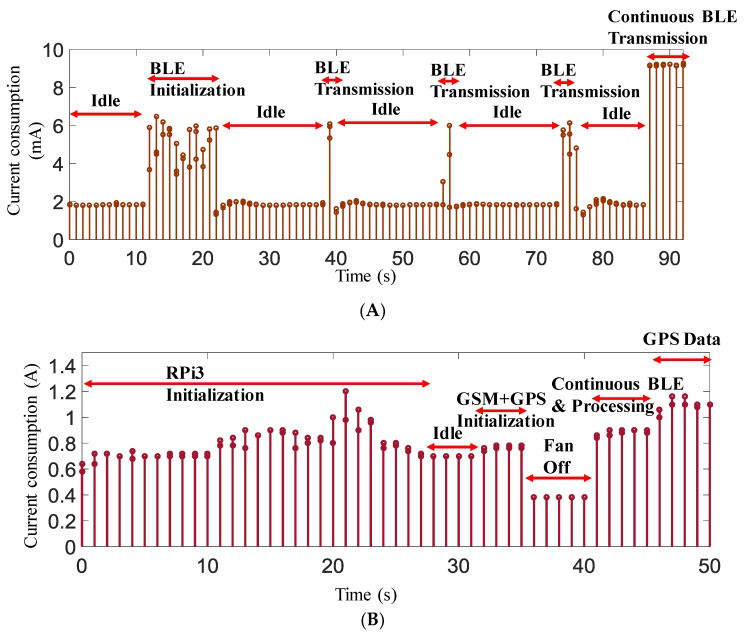
Current consumption in different operational scenarios for the wearable subsystem (**A**) and the RPI3 subsystem (**B**).

It was observed that the wearable subsystem drew the largest amount of current when the ECG data were acquired continuously, as shown in Figure 13A. In the case of burst transmission, approximately a 6 mA current was drawn. This subsystem continuously drained 2 mA, even if there was no BLE transmission. However, this system was designed to continuously transmit data every 20 ms and acquire data at every 2 ms interval. In continuous transmission mode, this subsystem drew 9.3 mA of current. Since the wearable subsystem would be running on battery, it was important to calculate the lifetime of the battery if it drew 9.3 mA of continuous current. The battery capacity could be calculated from the input current rating of the battery (1000 mAh in the prototype system) and the maximum load current (9.3 mA) of the circuit.

(7) $$Battery\;Life=\frac{Battery\;Capacity\left(mAh\right)}{Load\;Current\;in\;mill\;amps}*0.70$$

Equation (7) leads to 1000 mAh/9.3 mA × 0.7, i.e., approximately 75 h. Here, the factor of 0.7 (https://www.digikey.com/en/resources/conversion-calculators/conversion-calculator-battery-life) was used for external factors that could affect battery life. Therefore, it could be summarized that the wearable subsystem could be powered continuously for around three days from a 1000 mAh battery. However, the system would only be used by the driver during driving, therefore it would be expected that the daily driving time should not be more than 2–3 h for normal users unless the driver was driving a taxi. Battery should last up to a week for normal users if they do not use the system more than couple of hours in any day. Therefore, for the battery to last at least 24 h, the battery should be approximately 320 mAh in size.

The decision-making subsystem was designed with multiple components—RPi3, GPS, a GPRS/GSM shield, a cooling fan, and a BLE dongle. The initialization current consumption by RPi3 alone, RPi3 in idle mode and processing mode, fan consumption, and consumption of GSM and GPS modules and the complete system were recorded. Figure 13B shows the variation of current consumption by the decision-making subsystem at different scenarios. It is evident from Figure 13B that the RPi3 consumed around 1 A of current in its normal operational mode; however, part of this current was due to the cooling fan, as it was drawing approximately 0.3 A current. However, this subsystem took a maximum of 1.3 A when it was in the processing mode with continuous BLE transmission and GSM service and GPS data acquisition mode. The decision-making subsystem was powered using the vehicle’s cigarette lighter port, which was capable of supplying a constant 5 V supply with a maximum 2 A continuous current, which was much less than the current drawn by this subsystem. 

### 4.2. Performance Evaluation of MI Detection Algorithms

In the following section, the performance of linear and ML based algorithms are summarized.

**Figure 14:**
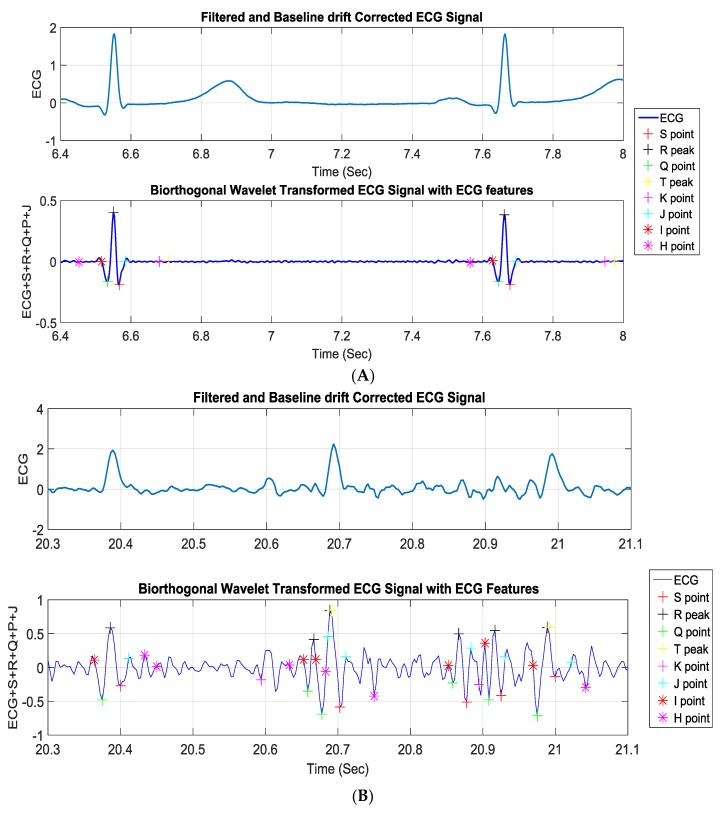
ECG parameters detection for normal subject (**A**) and effect of the motion artifact in the linear classification algorithm (**B**).

*Linear classification algorithm for MI detection*: The characteristic points of the ECG signal beside the R peak were extracted to fulfill the detection of myocardial infarction by the comparison of the isoelectric line with the ST segment, as shown in Figure 14A. Figure 14A demonstrates that the classical detection method worked reliably for noise free data. However, the linear classification and thresholding-based algorithm was not robust enough in the presence of movement artifacts induced in the ECG signals along with impulsive noises, and the algorithm failed in the noisy data (as shown in Figure 14B). Therefore, the machine learning based algorithm should be used for classifying ECG signals. 

*Machine Learning (ML) based MI detection algorithm:* All the features listed earlier were extracted, ROC analysis was performed for each feature, and AUC was calculated. Table 3 shows the AUC values for the feature extracted for ST-elevation and T-wave inversion from the different original t-domain, f-domain, and joint (t,f)-domain features for three different TFDs. All features that scored a minimum of 0.5 and above were considered to be useful to apply to classifiers. It was noticed that all the selected features fulfilled the requirement for any of the three distributions. Therefore, all the features were used for training and validation of 22 different machine learning models.

Computation of the ML algorithms validation accuracy was done using k-fold cross validation, where k was equal to five. There were five iterations, and the total accuracy was computed by obtaining the averages of the five accuracies and their standard deviations. Table 4 shows the accuracies resulting from classifying ST-elevation, and Table 5 shows the accuracies resulting from T-wave inversion for the three different (t,f)-distributions for the two best performing algorithms, the SVM and the k-nearest neighbors (KNN), where SVM outperformed KNN. The SVM training was selected to be a three-degree polynomial kernel function. The average accuracies of SVM for ST-elevation classification were 87.1 ± 7.77%, 85.3 ± 9.9%, and 97.4 ± 2.1% for WVD, SPEC, and EMBD distributions, respectively. The average accuracies for T-wave inversion classification were 78.0 ± 17.2%, 72.1 ± 3.09%, and 96.3 ± 0.66% for WVD, SPEC, and EMBD distributions, respectively.

It was quite evident that the EMBD outperformed the others in classifying ST-elevation and T-wave inversion in both the abnormal ECG wave classifications. Moreover, the standard deviation showed that the variation for different iterations was at minimum for the EMBD distribution; therefore, this distribution was more immune to noisy data. In the Python-based implementation, the t-domain, the f-domain, and the (t,f)-domain EMBD distributions were implemented for real-time classification. This was also revealed from the recall and the precision parameters. Both the recall and the precision were reasonable for reliable detection, and this was true for both positive and negative classifications, which was also reflected from the F-score.

## 5. Conclusions

In this study, we proposed and implemented a portable wearable ECG system for real-time heart attack detection. The hardware complexity was reduced by using off-the-shelf AD8232 AFE and a miniaturized microcontroller with built-in BLE. By using this device, the driver could keep track of his/her heart condition on a daily basis at low cost. Moreover, the immediate response on heart attack detection and alerting could help the driver to avoid road accidents and most likely save valuable lives. The linear classification algorithm is very fast in execution; however, it is unable to work with noisy ECG signals. However, the SVM algorithm with extended time-frequency features with the EMBD distribution showed highest accuracies of 97.4% and 96.3% for detecting ST-elevation and T-wave inversion, respectively. In addition, the proposed wearable device has lower power consumption, and therefore it is expected that the device can run for 24 h continuously with a 320 mAh battery. In summary, the device could contribute to excellent health monitoring and improve alerting services to the driver as well as to medical care-givers. In this manner, a driver could save himself/herself by avoiding fatal road accidents before losing his/her consciousness, and emergency medical services could approach the driver in a timely manner to provide required lifesaving medical procedures to avoid any life-threatening consequences. In the future, we would like to make the wearable device a wearable patch or a wearable smart watch to make it more feasible to the user. There will be some challenges in using a smart watch because of the hand movements; however, an adaptive motion artifact removal algorithm might enable a new way to modify this life-saving gadget.

## Figures and Tables

**Table 1:** Time-domain features with their mathematical expression.

Features	Mathematical Expression
Mean	$m\left(t\right)=\frac{1}{N}\sum_{n}x\left[n\right]$
Variance	$\sigma^{2}\left(t\right)=\frac{1}{N}\sum_{n}{\left(x\left[n\right]-m\left(t\right)\right)}^{2}$
Skewness	$\gamma \left(t\right)=\frac{1}{N\sigma^{3}\left(t\right)}\sum_{n}{\left(x\left[n\right]-m\left(t\right)\right)}^{3}$
Kurtosis	$k\left(t\right)=\frac{1}{N\sigma^{4}\left(t\right)}\sum_{n}{\left(x\left[n\right]-m\left(t\right)\right)}^{4}$
Coefficient of Variation	$C\left(t\right)=\frac{\sigma \left(t\right)}{m\left(t\right)}$

**Table 2:** Features extracted from the ECG traces.

Time-Domain Features	Frequency-Domain Features	Time-Frequency Features
▪ Mean ▪ Variance ▪ Skewness ▪ Kurtosis ▪ Coefficient of variance	▪ Spectral flux ▪ Spectral entropy ▪ Spectral flatness	▪ Combines all the pre-mentioned features ▪ Use Quadratic time-frequency distribution (QTFD) to find joint (t,f) representation: Winger-Ville Distribution (WVD) Spectrogram (SPEC) Extended Modified B-Distribution (EMBD)

**Table 3:** Results of the ROC analysis of t-domain, f-domain, and (t,f)-domain features for the ST-elevation detection.

Features	WVD		SPEC		EMBD	
	Original	Joint(t,f)	Original	Joint(t,f)	Original	Joint(t,f)
Mean	0.53	0.67	0.53	0.75	0.53	0.75
Variance	0.51	0.71	0.51	0.66	0.51	0.58
Skewness	0.68	0.57	0.68	0.67	0.68	0.57
Kurtosis	0.75	0.57	0.75	0.69	0.75	0.56
Coefficient of Variation	0.50	0.74	0.50	0.65	0.50	0.58
Spectral Flux	0.76	0.82	0.76	0.83	0.76	0.55
Spectral Flatness	0.54	0.71	0.54	0.79	0.54	0.68
Spectral Entropy	0.81	0.71	0.81	0.65	0.81	0.59

**Table 4:** Evaluation parameters in classifying ST-elevation.

Parameters/ML Algorithms	WVD		SPEC		EMBD	
	SVM	KNN	SVM	KNN	SVM	KNN
Recall (TPR)	92%	89%	89%	90%	99.1%	96.7%
FPR	11%	12%	14%	13%	1.7%	3.8%
Precision	89%	88%	86%	87%	98.3%	96.2%
F-score	90.5%	88.9%	87.5%	86%	98.7%	97%
Accuracy	87.1%	86.4%	85.3%	84.2%	97.4%	95.9%

**Table 5:** Evaluation parameters in classifying T-wave inversion.

Parameters/ML Algorithms	WVD		SPEC		EMBD	
	SVM	KNN	SVM	KNN	SVM	KNN
Recall (TPR)	86%	84%	83%	84%	98.5%	96.9%
FPR	19%	20%	22%	21%	1.3%	4.3%
Precision	81%	80%	78%	79%	97.8%	95.7%
F-score	83.4%	82.7%	75.9%	76.2	98.2%	96.6%
Accuracy	78%	76.3%	72.1%	74%	96.3%	95.1%

* WVD: Wigner-Ville distribution; SPEC: Spectrogram; EMBD: extended modified B-distribution; TPR: true positive rate; FPR: false positive rate.
